# Ultrathin Optics-Free Spectrometer with Monolithically Integrated LED Excitation

**DOI:** 10.3390/mi13030382

**Published:** 2022-02-27

**Authors:** Tuba Sarwar, Pei-Cheng Ku

**Affiliations:** Department of Electrical Engineering and Computer Science, University of Michigan, 1301 Beal Ave, Ann Arbor, MI 48109-2122, USA; tsarwar@umich.edu

**Keywords:** gallium nitride semiconductors, reflection spectroscopy, reconstructive spectrometers

## Abstract

A semiconductor spectrometer chip with a monolithically integrated light-emitting diode was demonstrated. The spectrometer design was based on a computational reconstruction algorithm and a series of absorptive spectral filters directly built in to the photodetectors’ active regions. The result is the elimination of the need to employ external optics to control the incident angle of light. In the demonstration, an array of gallium nitride (GaN) based photodetectors with wavelength selectivity generated via the principle of local strain engineering were designed and fabricated. Additionally, a GaN based LED was monolithically integrated. An optical blocking structure was used to suppress the LED-photodetector interference and was shown to be essential for the spectroscopic functionality. A proof of concept using a reflection spectroscopy configuration was experimentally conducted to validate the feasibly of simultaneously operating the LED excitation light source and the photodetectors. Spectral reconstruction using a non-negative least squares (NNLS) algorithm enhanced with orthogonal matching pursuit was shown to reconstruct the signal from the reflection spectroscopy. Optics-free operation was also demonstrated.

## 1. Introduction

Optical spectroscopy is one of the most widely used characterization techniques in science and engineering. Miniaturizing the optical spectrometer can allow for a portable and handheld system and lead to new opportunities for Internet of Things (IoT) and lab-on-a-chip applications [1]. These applications can have important impacts in the fields of chemistry, biomedicine, food engineering, planetary exploration, point-of-care services, and the life sciences [2,3,4,5,6,7,8,9,10,11]. A spectrometer is a highly complex system consisting of optical, mechanical, and image processing units. Miniaturizing such a system is a nontrivial task involving the ability to integrate multiple material platforms and careful planning of performance tradeoffs, such as spectral resolution, sensitivity, system size, and cost. Among various approaches, spectrometers based on reconstructive algorithms [12] shift the complexity of processing spectral information from physical components to software computations [13,14,15,16,17,18,19,20,21,22,23,24,25]. With the steady growth of computational power per watt-dollar, this approach has become increasingly promising in constructing an extremely compact spectroscopic system.

A reconstructive spectrometer consists of a series of photodetectors with different spectral responses $R_{k}\left(\lambda \right)$, where *k* denotes the *k*th detector and λ is the optical wavelength. For an unknown spectrum S(λ), the photocurrent generated by the *k*th detector is given by $I_{k}=R_{k}\left(\lambda \right)S\left(\lambda \right)$. With a set of known $R_{k}\left(\lambda \right)$, one can reconstruct the unknown spectrum S(λ) by inverting the above equation using a computational algorithm, e.g., the non-negative least squares (NNLS) method. The spectral resolution is determined by the size of the $R_{k}\left(\lambda \right)$ matrix and how randomly each photodetector’s spectral response relates to another. Ideally, there should be no correlations between any two photodetectors’ responses and the number of photodetectors should be equal to the spectrometer’s spectral range divided by the desired spectral resolution. For many applications, however, a reconstructive spectrometer can generate useful information even working as an underdetermined system. For example, the locations of a spectrum’s peaks and the relative intensities between them are often sufficient information. Moreover, the reconstruction algorithms can be enhanced by adding constraints that can either be improved over time via learning or be constructed based on the specific characteristics of the application of interest [26,27,28]. This is highly desirable for many applications targeted by a miniaturized spectrometer.

A conventional semiconductor-based photodetector’s spectral response cannot be easily tuned. Therefore, a reconstructive spectrometer has often been implemented with a series of filter/detector combinations. However, dispersive spectral filters are highly sensitive to the incident angle of light, requiring additional collimation optics which add to a system’s bulkiness and weight. Recently, absorptive filters, either standalone or built into the photodetectors, have been successfully integrated into a reconstructive spectrometer [13,14,18,21,29]. Absorptive filters’ spectral responses have a weak dependence on the incident angle of light [22]. As a result, collimation optics either become optional or can be replaced by other imaging optics to enable a spectrometer array for hyperspectral imaging. 

Most spectroscopic applications require not only a detection system but also an excitation light source to provide a reference in reflection and transmission spectroscopy or to excite the analyte in fluorescence spectroscopy. Chip-scale integration of an LED (light-emitting diode) light source is an important part of miniaturizing a spectroscopic system. However, very few works have been carried out to address this need [5,30,31,32,33]. The main challenges include the heterogeneous integration of different material platforms for the light source and detectors, the already considerable size of the optical detection system preventing further integration of the light source, and interference between the light source and the spectral signal. In this work, we successfully demonstrated monolithic integration of LEDs and an array of wavelength-selective photodetectors. We experimentally validated the feasibility by showing the negligible interference of the on-chip LED due to the portion of light directly leaked to the detector array. We also showed an example of reflection spectroscopy using the on-chip LED and a notch filter to simulate an analyte absorbing at a specific wavelength. 

## 2. Materials and Methods

The spectrometer design consists of an array of GaN-based wavelength-selective photodetectors [21]. When the strained InGaN quantum well active region is made into a nanopillar geometry, the strain relaxation shifts the emission wavelength of the quantum well to a short wavelength [34]. The strain relaxation is nonuniform and decreases from the edge to the center. As GaN exhibits a large piezoelectric polarization due to the in-plane strain, the strain relaxation leads to the change of the bandgap and the absorption cutoff wavelength. The amount of wavelength shift depends on the nanopillar’s diameter and the emission wavelength of the original quantum well. In this work, the sample’s epitaxial structure includes five periods of InGaN/GaN quantum wells sandwiched between a GaN pn junction. The room temperature (sample uncooled) electroluminescence peaked at 590 nm and can be tuned between 480 and 590 nm depending on the nanopillar diameter.

**Figure 1:**
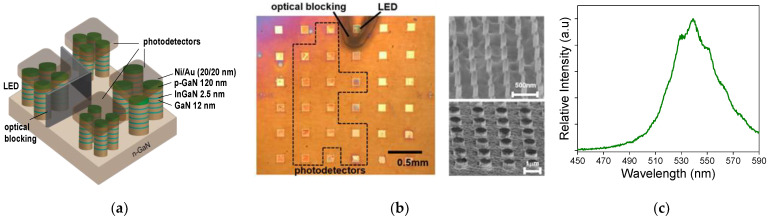
(**a**) Schematic of the proposed spectrometer chip with a monolithically integrated LED. As shown in the illustration, there are three photodetectors and one LED separated by an optical blocking structure. The three photodetectors exhibit different spectral responses due to the difference of the nanopillar diameter. (**b**) The optical image of the as-fabricated spectrometer chip with a monolithically integrated LED light source. Each yellow square is a nanopillar array with a chip area of 100 µm × 100 µm. The nanopillar diameters of the 13 arrays enclosed by the dashed line vary from 55 nm through thin film (see Section 3 for the diameter values). The black-colored structure is the enamel-coated PET optical blocking structure to suppress the direct capture of the LED emission by the photodetectors. The edge-to-edge spacing between two arrays is 0.3 mm. The two scanning electron micrographs show two nanopillar arrays after etching but before planarization. The diameters of the nanopillars are 80 nm and 800 nm for the images on the top and bottom, respectively. (**c**) The emission spectrum of the on-chip LED (biased at 8V) measured by Ocean Optics HR2000. The LED device is also a nanopillar array with a diameter of 200 nm.

Figure 1a shows the device schematic. The device consists of an array of photodetectors, each constructed based on an array of GaN nanopillars comprising InGaN/GaN multiple quantum wells. It also has an LED which was made of GaN nanopillars. The nanopillar’s diameter was varied to control emission and absorption properties. The sample was grown by metal–organic chemical vapor deposition (MOCVD) on a c-plane sapphire substrate. After growth, the sample was patterned using electron-beam lithography into a series of nanopillar arrays. Each array is either a photodetector or LED depending on the bias. Each array occupied a chip area of 100 µm by 100 µm. All nanopillars in the same array have the same diameter. The spacing between two adjacent nanopillars was 250 nm for smaller nanopillar diameters and 2 µm for larger ones. The nanopillars were formed using a two-stage etching process, first using inductively coupled plasma-reactive ion-etching (ICP-RIE) followed by an anisotropic wet etch in a 2% diluted KOH solution (AZ-400). Chromium was used as the etch mask. The wet etch step created a vertical sidewall, allowing the nanopillar diameter to be better controlled. The etching was stopped once we cleared the quantum well region. The resulting nanopillar height was 260 nm. After etching, a conformal 50 nm thick SiN_x_ layer was deposited to form electrical insulation around the nanopillars. The sample was then finished with planarization using SiO_2_ and electrode deposition (Ni–Au for p-contact and Ti–Au for n-contact). 

Two sets, each containing a total of 14 nanopillar arrays were fabricated along with a few additional test structures. The optical image of the as-fabricated sample and the scanning electron micrographs of two of the arrays immediately after the patterning step are shown in Figure 1b. To choose the nanopillar diameters, we aim to distribute the absorption cutoff wavelengths of the nanopillar arrays evenly across a wide wavelength range. Previously, using a simple one-dimensional solid mechanics model which modeled the InGaN quantum well with a linear chain of atoms whose positions were determined from the balance of the strain relaxation due to the nanopillar geometry and the intrinsic compressive strain due to the lattice mismatch. The result is that the emission wavelength of each nanopillar array follows an exponential dependence 1−sinhκD/2 on the nanopillar’s diameter D, where κ describes the material’s elastic property [34]. The emission wavelength of each nanopillar array was previously shown to be identical to the absorption cutoff wavelength at a zero bias [21]. To this end, we varied the nanopillar diameter from 55 to 100 nm with a 5 nm increment and from 100 nm to thin film with a larger increment to even out the emission wavelength distribution across the blue–green–orange range. 

**Figure 2:**
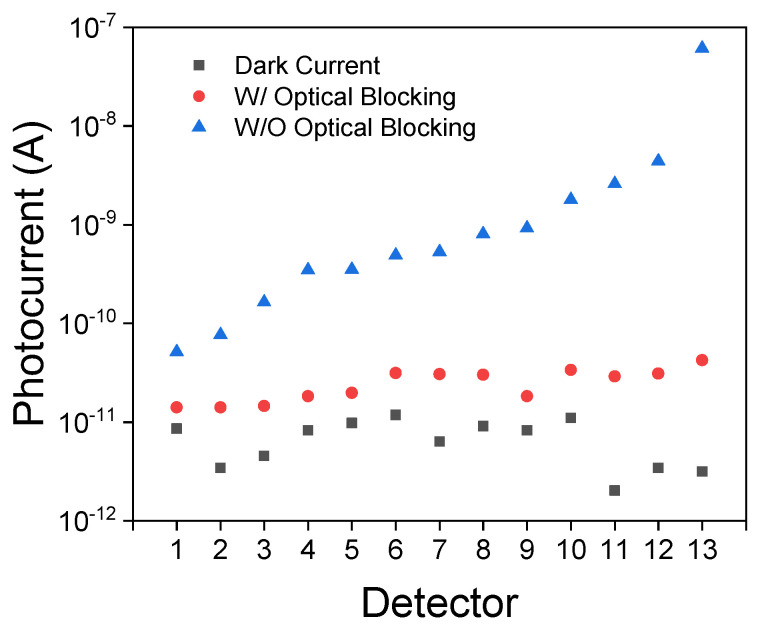
The photocurrents measured from the 13 photodetectors using a semiconductor parameter analyzer (Keithley 4200) under various scenarios: the dark currents measured when the on-chip LED was electrically disconnected; the photocurrents with and without the optical blocking structure integrated on the LED (shown in Figure 1a) which was biased at a constant 8V. LED emission reflected from an off-chip object is negligible. As a result, the photocurrents measured were the result of LED emissions being directly captured by the photodetectors along a direct path on the chip. With optical blocking, the photocurrent due to the LED interference can be considerably suppressed to be within the same order of magnitude as the dark current.

One of the 14 nanopillar arrays was chosen to operate in the LED mode with a forward bias applied. The emission spectrum was measured by Ocean Optics HR2000 and is shown in Figure 1c. As shown in Figure 1b, the LED is placed adjacent to the photodetector array. The spacing between each array is 0.3 mm. Due to the proximity, the LED emission can be directly captured by the photodetectors. This interference can overwhelm the spectroscopic signal, which is likely to be much weaker than the emission along a direct path from the LED to the photodetectors. To address this challenge, we fabricated an optical blocking structure using polyethylene terephthalate (PET). We first coated the PET with black enamel. We then shaped the PET sheet into a wall-like structure and attached it to the sample surrounding the LED. Figure 2 shows that the PET optical blocking layer successfully suppressed the LED interference. The photocurrents due to the direct LED emission being captured by the photodetectors were significantly reduced to be within the same order of magnitude as the dark currents measured with the LED turned off.

## 3. Results

**Figure 3:**
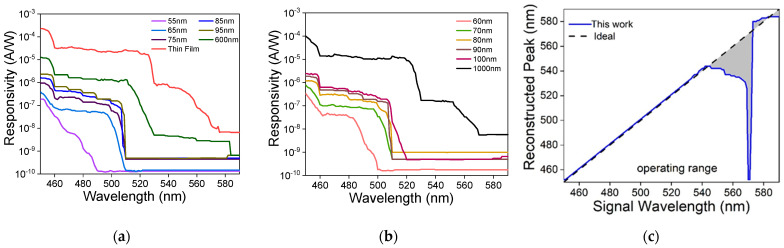
(**a**,**b**) The responsivities of the 13 photodetectors measured at a zero bias using a tunable monochromatic light source (adapted from [22]). The optical power at the sample was characterized separately using an integration sphere. The photocurrent was measured using a semiconductor parameter analyzer (Keithley 4200). The legend shows the nanopillar’s diameter in each photodetector. (**c**) Determination of the spectrometer’s operating range using an NNLS algorithm with a series of delta-function spectra between 450 nm and 590 nm. The shaded area from 541 to 590 nm indicates the wavelength range in which the reconstruction has a large error. The result implies an operating range from 450 nm through 540 nm.

Unlike a filter-based spectrometer, the reconstructive spectrometer’s operating range is largely determined by the wavelength range in which the photodetectors’ responses do not have a strong correlation. To determine the operating wavelength range of our device, we first measured each photodetector’s responsivity between 450 and 590 nm with a 1 nm spectral resolution. All photodetectors were kept at a zero bias. The results are shown in Figure 3a,b. The responsivity shown uses a standard definition for a silicon photodetector and accounted for all the light not absorbed when passing between the nanopillars and ~88% not absorbed when passing through the quantum wells. A stronger absorption, e.g., by increasing the nanopillar array’s fill factor, can further enhance the responsivity. 

Once the responsivities of all 13 photodetectors were obtained, we can determine the operating range of the spectrometer by performing the NNLS algorithm with a series of delta-function spectra, i.e., spectra with only a finite value at a specific wavelength and zeros everywhere else. The result is shown in Figure 3c. The reconstruction is accurate between 450 and 540 nm. Although the emission wavelength of the photodetector ranges from 480 through 590 nm, the operating window is not the same. The photodetectors’ responses beyond 540 nm are not sufficiently random, which results in a large reconstruction error between 540 and 590 nm. This can be remedied by increasing the responsivities of all photodetectors [22]. In contrast, although all photodetectors absorb strongly between 450 and 480 nm, the correlation between their responsivities is weak enough to still lead to an accurate reconstruction. 

**Figure 4:**
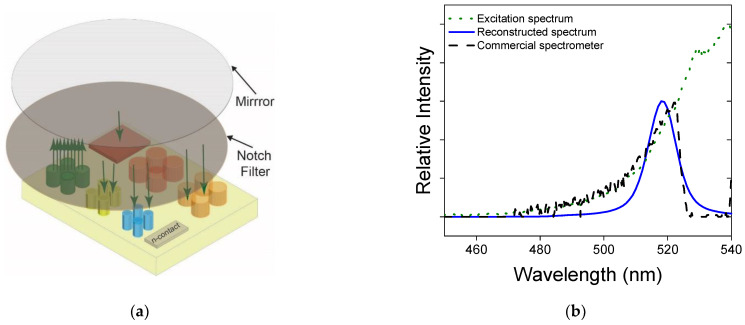
(**a**) The experimental configuration to demonstrate the feasibility of the monolithically integrated LED light source in reflection spectroscopy. A notch filter was used to modify the LED emission. Together with the mirror, the reflected spectrum simulated an analyte absorbing around 533 nm with an absorption linewidth of 17 nm. The distance between the mirror and the chip is 4 cm. No other optics other than the two shown were used. (**b**) The LED emission (green dotted curve), the reflected spectrum (black dashed curve) just above the chip captured by Ocean Optics HR2000, and the reconstructed spectrum (blue solid curve) using an OMP-enhanced NNLS algorithm.

Next, we demonstrated the feasibility of the monolithically integrated LED light source in the configuration of reflection spectroscopy. We placed a mirror approximately 4 cm from the sample, as shown in Figure 4a. We then inserted a notch filter (Thorlabs NF-533-17) with a center wavelength of 533 nm and a linewidth of 17 nm between the mirror and the sample. No other optics were used. In the experiment, the LED emission emanated from the sample passed through the notch filter, was reflected from the mirror, and passed through the filter another time before being absorbed by the photodetectors. The returned spectrum was measured by a commercial spectrometer and shown by the dashed curve in Figure 4b. 

To reconstruct the reflected spectrum, we used an NNLS algorithm enhanced by the orthogonal matching pursuit (OMP) method to address a full range of spectral components simultaneously at multiple photodetectors [21]. We used a Gaussian basis with a 12 nm linewidth and a maximum iteration value of 141. The reconstructed spectrum is shown in Figure 4b with the blue solid curve which reasonably matches the spectrum measured by a commercial spectrometer (black dashed curve). The spectral reconstruction in this work is an underdetermined problem with only 13 photocurrent values available. As a result, only an approximate instead of an exact spectrum can be reconstructed. 

## 4. Discussion

The reconstructive spectrometer chip designed, fabricated, and characterized in this work used a series of wavelength-selective photodetectors which exhibited a very weak dependence on the incident angle of light. As a result, collimation optics were not necessary for the functionality of the device. It was evident that the responsivities used in the spectral reconstruction in Figure 4b were measured using a completely different illumination profile. The responsivities were measured using a monochromatic light focused onto the sample while the reflected spectrum in Figure 4 diverged significantly due to the incoherent nature of the LED emission. The results highlighted an important advantage of the reconstructive spectrometer using absorptive spectral filters, which in our case were built in as part of the photodetectors. The optionality of the collimation optics can enable a miniaturized spectrometer in an ultrathin form factor. One can also opt to integrate focusing lenses to increase the signal-to-noise level and/or to easily enable an array of spectrometers on the chip for hyperspectral imaging.

The spectroscopic operating range in this work can be expanded by reducing the correlation between the photodetectors’ responses, e.g., by enhancing the absorption in the long-wavelength region. When some photodetectors’ responsivities are very low, they effectively become non-participating and correlated, as 0 and 0 are always correlated. The enhanced absorption can allow more photodetectors to participate in the reconstruction algorithm. This was shown in [22].

The optical blocking in this work was fabricated using an enamel-coated PET sheet manually attached to the spectrometer chip. A more scalable approach can be to use a MEMS (microelectromechanical system) process. For example, when patterning the nanopillar arrays, one can keep a checkerboard pattern of GaN unetched. These wall-like structures can then be heightened using PDMS (polydimethylsiloxane) which can be subsequently coated with a thin metal layer to be rendered light-blocking. As the LED is simply a forward-biased photodetector in our design, a photodetector array separated by optical blocking structures can enable a reconfigurable excitation spectrum for more versatile applications. 

## 5. Conclusions

In summary, we designed, fabricated, and demonstrated the feasibility of an optics-free spectrometer chip with a monolithically integrated LED light source. With 13 photodetectors, we successfully achieved spectroscopic functionalities in the wavelength range between 450 nm and 540 nm. We also demonstrated the feasibility of reflection spectroscopy using the on-chip LED source and photodetectors simultaneously. The optical blocking structure successfully suppressed the direct capture of the LED emission by the photodetectors, which were as close as 0.3 mm from the LED. Finally, we showed that the spectrometer chip in this work did not require any external optics for its functionality. Therefore, the proposed spectrometer design can potentially enable a miniaturized spectroscopic system in an ultrathin film platform which can enable new opportunities, e.g., a wearable spectrometer chip to monitor physiological conditions in real time. 

## Data Availability

The data presented in this study are available on request from the corresponding author.
